# P2-Na_0.6_[Cr_0.6_Ti_0.4_]O_2_ cation-disordered electrode for high-rate symmetric rechargeable sodium-ion batteries

**DOI:** 10.1038/ncomms7954

**Published:** 2015-04-24

**Authors:** Yuesheng Wang, Ruijuan Xiao, Yong-Sheng Hu, Maxim Avdeev, Liquan Chen

**Affiliations:** 1 Key Laboratory for Renewable Energy, Beijing Key Laboratory for New Energy Materials and Devices, Beijing National Laboratory for Condensed Matter Physics, Institute of Physics, Chinese Academy of Sciences, Beijing 100190, China; 2 Bragg Institute, Australian Nuclear Science and Technology Organisation, Locked Bag 2001, Kirrawee DC NSW 2232, Australia

## Abstract

Most P2-type layered oxides exhibit Na^+^/vacancy-ordered superstructures because of strong Na^+^–Na^+^ interaction in the alkali metal layer and charge ordering in the transition metal layer. These superstructures evidenced by voltage plateaus in the electrochemical curves limit the Na^+^ ion transport kinetics and cycle performance in rechargeable batteries. Here we show that such Na^+^/vacancy ordering can be avoided by choosing the transition metal ions with similar ionic radii and different redox potentials, for example, Cr^3+^ and Ti^4+^. The designed P2-Na_0.6_[Cr_0.6_Ti_0.4_]O_2_ is completely Na^+^/vacancy-disordered at any sodium content and displays excellent rate capability and long cycle life. A symmetric sodium-ion battery using the same P2-Na_0.6_[Cr_0.6_Ti_0.4_]O_2_ electrode delivers 75% of the initial capacity at 12C rate. Our contribution demonstrates that the approach of preventing Na^+^/vacancy ordering by breaking charge ordering in the transition metal layer opens a simple way to design disordered electrode materials with high power density and long cycle life.

## Abstract (web summary)

Sodium-containing layered oxides are promising battery cathodes, but their performance suffers from the formation of sodium ion-vacancy ordered superstructures. Here, the authors present a P2-Na_0.6_[Cr_0.6_Ti_0.4_]O_2_ layered oxide with disordered cations, leading to high battery performance.

## Graphical abstract

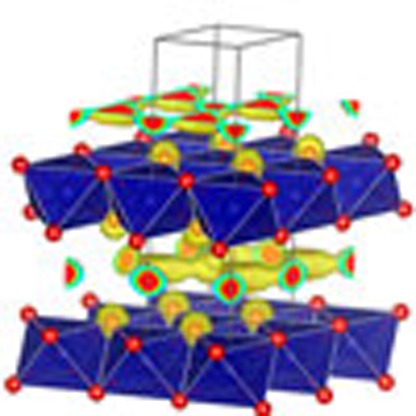

Layered oxides have attracted great attention because of their unique low-dimensional structure and physical/chemical properties. In particular, they are a class of important cathode materials for rechargeable batteries. Among them, LiCoO_2_ is still the most successful high-energy-positive electrode material for lithium-ion batteries^1^. In sodium-ion batteries showing worldwide interest for large-scale energy storage recently^2^,^3^,^4^,^5^,^6^,^7^, Na_*x*_CoO_2_ is known as the first layered oxide investigated as a Na intercalation host^8^. In this compound, depending on sodium content and temperature an interplay between Na^+^–Na^+^ and Na^+^–Co^3+/4+^ electrostatic interactions coupled with Co^3+/4+^ charge ordering results in the formation of numerous Na^+^/vacancy-ordered superstructures. Such ordered superstructures have distinct voltage plateaus in electrochemical curves^9^, lower Na^+^ ion diffusion coefficient (by up to two orders of magnitude^10^) and even reduced dimensionality of ionic transport^11^. The combination of these phenomena in turn results in rapid capacity-fading during cycling. Such a common behaviour was also observed in other layered oxides with brucite-type octahedral (MO_6_) layers, for example, Na_*x*_MnO_2_ (ref. 12, 13, 14, 15), Na_*x*_VO_2_ (ref. 16), Na_2/3_Ni_1/3_Mn_2/3_O_2_ (ref. 17), Na_2/3_Co_2/3_Mn_1/3_O_2_ (ref. 18), and so on. The goal of this work was to understand the conditions leading to Na^+^/vacancy ordering and potentially find a way to prevent it. We focused on P2-type layered oxides, which are known to be especially favourable for high Na^+^ ion diffusion in trigonal prismatic interlayer space.

In general, there are three types of ordering in P2-Na_*x*_[M1,M2]O_2_-layered oxides: transition metal ordering, charge ordering and Na^+^/vacancy ordering, which affect each other (Table 1). In the case of transition metal ordering, we found that as expected it is mainly controlled by the difference in the ionic radii of M1 and M2, as illustrated by a large number of P2-layered oxides^6^,^8^,^9^,^12^,^13^,^14^,^15^,^16^,^17^,^18^,^19^,^20^,^21^,^22^,^23^,^24^,^25^,^26^,^27^,^28^,^29^,^30^,^31^,^32^,^33^,^34^,^35^. Large difference in ionic radii is favourable for forming ordered arrangement while small difference tends to form disordered arrangement. In line with the general trend well established in crystal chemistry, we found the critical difference ionic radii to be ∼15%. If the radii difference is higher than 15% and M1/M2 content is close to a rational ratio, then an ordered structure is expected; otherwise M1 and M2 are disordered. Occurrence of charge ordering is determined by the redox potentials (that is, Fermi level) of M1 and M2. Small difference is favourable for charge ordering even if M1 and M2 are chemically disordered, for example, Na_x_[Mn_y_Co_1-y_]O_2_ (*y*<0.5; Table 1), whereas large difference tends to form charge disordering. Finally, Na^+^/vacancy ordering and charge ordering are coupled to each other. There is no charge ordering without Na^+^/vacancy ordering and *vice versa*.

Here we intentionally chose the transition metal ions with very similar ionic radii and substantially different redox potential versus Na, such as Cr^3+^ and Ti^4+^ in expectation to build the P2-layered oxide free of Na^+^/vacancy ordering. Disordered arrangement of Cr^3+^ and Ti^4+^ in the transition metal layers prevents M^3+/4+^ charge ordering such as observed in Na_*x*_CoO_2_ (ref. 9) and Na_*x*_VO_2_ (ref. 16) that in turn prevents Na^+^/vacancy ordering. Using neutron powder diffraction (NPD) we show that P2-Na_0.6_[Cr_0.6_Ti_0.4_]O_2_ synthesized through a simple solid-state reaction is Na^+^/vacancy-disordered at any sodium content in the studied range of *x*=0.33–1.0 in Na_*x*_[Cr_0.6_Ti_0.4_]O_2_ and temperature from 3 to 1,073 K that leads to its very high ionic conductivity and ability to withstand high charge/discharge rates.

## Results

### Crystal structure of P2-Na_0.6_[Cr_0.6_Ti_0.4_]O_2_

This oxide was synthesized by a simple solid-state reaction using precursors of Na_2_CO_3_, Cr_2_O_3_ and TiO_2_. The inductively coupled plasma (ICP) result confirms the composition of Na_0.605_[Cr_0.6_Ti_0.4_]O_2_. The morphology of the sample was investigated with scanning electron microscopy (SEM). As shown in the inset of Supplementary Fig. 1, the distribution of particle size of the as-prepared sample is in the range of 1–4 μm. Sample phase purity was verified using powder X-ray diffraction. All the diffraction peaks were found in good agreement with the JCPDS No. 52-537 (ref. 36) that indicated a pure P2 phase material. The crystal structure of the material was further studied in detail using NPD. Crystal structure of the P2-type is built of brucite-type edge-sharing octahedral layers containing transition metals with trigonal prismatic interlayer space containing sodium^37^. There are two inequivalent trigonal prismatic positions available for Na atoms: sharing faces and edges with transition metal octahedra, designated in the following text Na1 and Na2, respectively. For the title composition Na_0.6_[Cr_0.6_Ti_0.4_]O_2_, two questions of crucial importance were whether Cr and Ti are ordered in the octahedral layers and whether Na atoms are ordered over Na1 and Na2 prisms. Both aspects were studied in detail using NPD, which was clearly a technique of choice in this case. First, in contrast to X-ray diffraction, NPD is extremely sensitive to Cr and Ti distribution because of an opposite sign of the neutron-scattering lengths (3.635 and −3.370 fm, respectively). Second, again in contrast to X-ray diffraction, in the case of random distribution of Cr and Ti the contribution of transition metals to the total diffraction pattern is rather small (b_coh_(Cr_0.6_Ti_0.4_)=0.833 fm) that makes NPD more sensitive to the contribution from the sodium sublattice (b_coh_(Na_0.6_)=2.18 fm).

**Figure 1:**
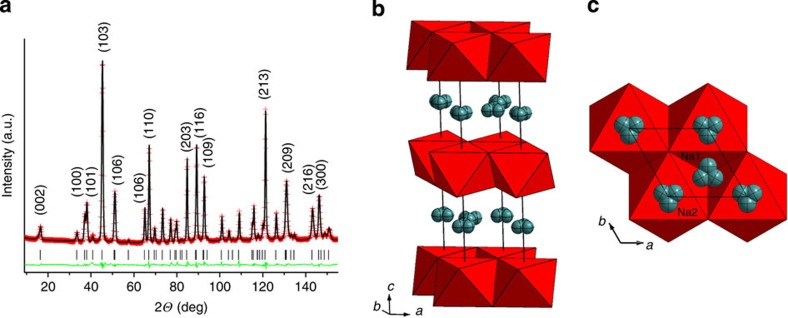
Structure of P2-Na_0.6_[Cr_0.6_Ti_0.4_]O_2_. (**a**) The Rietveld plot for P2-Na_0.6_[Cr_0.6_Ti_0.4_]O_2_ refined against NPD data collected at 300 K. The red crosses and black and green solid lines indicate the observed and calculated patterns and their difference, respectively. The tick marks indicate the position of the diffraction peaks. *R*_p_=2.08%, *R*_wp_=2.73%. (**b**,**c**) General view of the crystal structure and view along the *c* axis showing splitting of Na1 and Na2 sites from the centres of prisms.

At room temperature the high-quality NPD data unambiguously revealed absence of supercell diffraction peaks and therefore a high symmetry model (space group P6_3_/mmc no. 194) with a unit cell (*a*∼3 Å, *c*∼11 Å) and random distribution of Cr/Ti and Na over a single octahedral site and two trigonal prismatic sites, respectively, was successfully used to analyse the data (also see Supplementary Fig. 2). The absence of Cr/Ti ordering is not surprising as their ionic radii are extremely close (0.615 and 0.605 Å, respectively)^38^. Furthermore, in the beginning, we used a model with Na atoms placed in the centres of prisms, that is, 2*c* and 2*b* Wyckoff sites, respectively. However, Difference Fourier map revealed clearly off-centre nuclear density, which could not be adequately modelled by anisotropic thermal parameters because of the point symmetry of the sites. Therefore, both Na1 and Na2 positions were split into 6-h (*x*, 2*x*, 1/4) sites that significantly improved refinement quality. The *R*_p_ and *R*_wp_ reduced from 2.30% and 2.99% to 2.08% and 2.73%, respectively, with only two additional variables, which is certainly statistically significant^39^. Such sodium site splitting was previously proposed in several structural studies of various P2-type materials^40^,^41^,^42^. The Rietveld plot and crystallographic parameters are presented in Fig. 1 and Supplementary Tables 2 and 3, respectively. We believe that similar off-centre Na location may exist in all the other P2-Na_*x*_[M1,M2]O_2_ compositions with M1=V–Ni and M2=Ti, Li, Mg, and so on because of the asymmetric local coordination of Na by M1 and M2. Uncompensated electrostatic repulsion displaces Na^+^ from prism centres.

### Sodium storage performance

**Figure 2:**
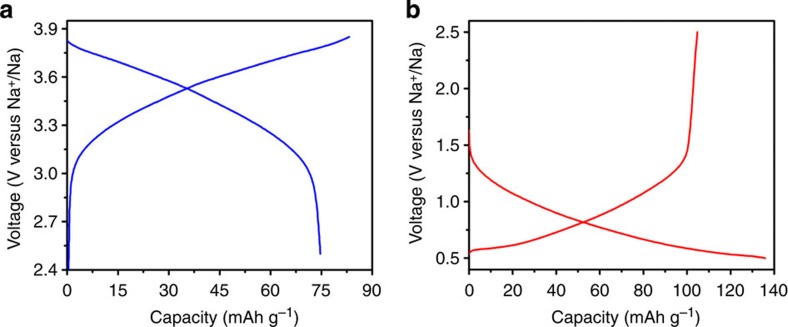
Typical first charge/discharge profiles of P2-Na_0.6_[Cr_0.6_Ti_0.4_]O_2_ electrodes in the NaPF_6_-based electrolyte. (**a**) P2-Na_0.6_[Cr_0.6_Ti_0.4_]O_2_ tested as positive electrode in the voltage range of 2.5–3.85 V and (**b**) P2-Na_0.6_[Cr_0.6_Ti_0.4_]O_2_ tested as negative electrode in the voltage range of 0.5–2.5 V.

**Figure 3:**
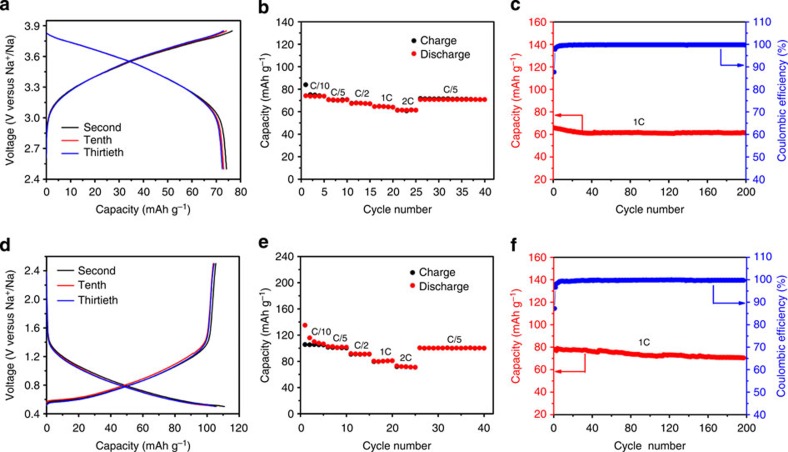
Sodium storage performance of P2-Na_0.6_[Cr_0.6_Ti_0.4_]O_2_ electrodes in the NaPF_6_-based electrolyte. (**a**) The second, tenth and thirtieth charge/discharge profiles at a current rate of C/10 (7.6 mA g^−1^) in the voltage range of 2.5–3.85 V versus Na^+^/Na. (**b**) Rate capability. The capacity versus cycle number at various current rates. (**c**) Long-term cycling performance. The capacity and Coulombic efficiency versus cycle number at a current rate of 1C. (**d**) The second, tenth and thirtieth charge/discharge profiles at a current rate of C/10 (11.2 mA g^−1^) in the voltage range of 0.5–2.5 V versus Na^+^/Na. (**e**) Rate capability. The capacity versus cycle number at various current rates. (**f**) Long-term cycling performance. The capacity and Coulombic efficiency versus cycle number at a current rate of 1C.

The sodium storage performance of P2-Na_0.6_[Cr_0.6_Ti_0.4_]O_2_ in sodium half cells using 0.8 M NaPF_6_ in ethylene carbonate (EC)/diethyl carbonate (DEC) as the electrolyte is shown in Figs 2 and 3. Since the valence state of Cr in this compound is 3+, it can be oxidized to 4+ on Na deintercalation, enabling it as a possible positive electrode. Meanwhile, the valence state of Ti in this compound is 4+; it can be reduced to 3+ on Na intercalation, making it a possible negative electrode. Figure 2 shows the typical first charge and discharge curves of Na_0.6_[Cr_0.6_Ti_0.4_]O_2_ electrodes in the voltage ranges of 2.5–3.85 and 0.5–2.5 V, respectively. It can be seen that all the charge and discharge curves show smooth slopes without plateaus, indicating the sodium deintercalation and intercalation through a single-phase reaction. This is further supported by the *in situ* XRD results as discussed later. This behaviour is very different from some other P2-type electrodes such as Na_*x*_CoO_2_, Na_*x*_VO_2_, where multiple plateaus were observed during sodium deintercalation/intercalation, which is related to the formation of Na^+^/vacancy and charge-ordered superstructures^9^,^16^. In the case of Na_0.6_[Cr_0.6_Ti_0.4_]O_2_, the disordered distribution of Cr and Ti in the transition metal layer as discussed above is responsible for such sodium storage mechanism.

Interestingly, the average Na storage voltage for the positive electrode is *ca.* 3.5 V, which is much higher than that of O3-type NaCrO_2_ (*ca.* 3 V) with the same Cr^3+^/Cr^4+^ redox couple^43^. This is likely related to the different sodium contents in Na_0.6_[Cr_0.6_Ti_0.4_]O_2_ and NaCrO_2_ samples. The reversible discharge capacity is ∼74 mAh g^−1^, corresponding to 0.26 Na deintercalation/intercalation per formula unit. The rate capability of the Na_0.6_[Cr_0.6_Ti_0.4_]O_2_ electrode was also tested in a sodium-ion battery. It can be seen from Fig. 3b that the reversible capacities are 74, 70, 67, 64 and 61 mAh g^−1^ at constant current rates of C/10, C/5, C/2, 1C and 2C, respectively. The capacity retention at 1C is 82% of the initial capacity. The long-term cyclic stability of this material is also excellent at current rates of 0.1C and 1C as shown in Fig. 3a,c. The Na_0.6_[Cr_0.6_Ti_0.4_]O_2_ electrode exhibits over 200 cycles with capacity retention of 94% at a current rate of 1C. The Coulombic efficiency after initial cycles reaches nearly 99.4%.

For the negative electrode side, the average sodium storage voltage is *ca.* 0.8 V, which is similar to P2-Na_0.66_[Li_0.22_Ti_0.78_]O_2_ (ref. 22). In the Na_0.6_[Cr_0.6_Ti_0.4_]O_2_, the available vacancy in the alkali layer will allow 0.4 Na intercalation into the host, corresponding to a capacity of 112 mAh g^−1^, which is in good agreement with the observed reversible charge capacity of 105 mAh g^−1^. The Coulombic efficiency of the first cycle is ∼80% in the NaPF_6_-based electrolyte. This low Coulombic efficiency is because of the reduction of the used electrolyte to form the solid electrolyte interphase (SEI) layer on the surface of the electrode. It is noted that when a NaClO_4_-based electrolyte was used, an even much lower Coulombic efficiency of 54% was observed as shown in Supplementary Fig. 3, indicating a serious decomposition of the electrolyte. It was found that the thickness of the formed amorphous SEI layer on the electrode from the NaPF_6_-based electrolyte is much thinner than that from the NaClO_4_-based electrolyte (Supplementary Fig. 4), which coincides with the observation of irreversible capacity loss in different electrolytes. Nevertheless, a similar Na storage performance in terms of rate capability and long cycle stability was also achieved in the NaClO_4_-based electrolyte (Supplementary Fig. 3). In terms of rate performance, it displays slightly lower rate capability compared with that of the positive electrode as shown in Fig. 3e and Supplementary Fig. 5. This is likely related to smaller number of available vacancies for the Na content (*x*=0.6–1) in the Na_*x*_[Cr_0.6_Ti_0.4_]O_2_ negative electrode, resulting in slower Na^+^ ion diffusion because of the strong repulsive electrostatic interactions among Na^+^ ions. Again, it can be seen from Fig. 3d,f that the material presents very stable cycling performance at low and high current rates. After 200 cycles at 1C rate, the capacity retention is 90.4% (also see Supplementary Fig. 6). The superior Na storage performance from both positive and negative electrodes originates from the peculiar cation-disordered feature in Na_0.6_[Cr_0.6_Ti_0.4_]O_2_. This performance is much better than that of other P2-type oxides with ordered structure^6^,^9^,^13^,^15^,^16^,^17^,^18^,^20^,^24^.

**Figure 4:**
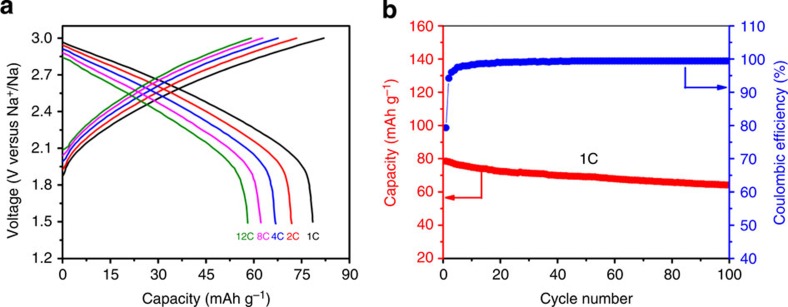
Full-cell performance of P2-Na_0.6_[Cr_0.6_Ti_0.4_]O_2_ as both positive and negative electrodes in the NaPF_6_-based electrolyte. (**a**) Discharge profiles of Na_0.6_[Cr_0.6_Ti_0.4_]O_2_/Na_0.6_[Cr_0.6_Ti_0.4_]O_2_ sodium-ion full cell at various rates. (**b**) The capacity and Coulombic efficiency versus cycle number of the full cell at 1C rate. The capacity was calculated based on the mass of negative electrode.

On the basis of above results, a sodium-ion full cell was also demonstrated using P2-Na_0.6_[Cr_0.6_Ti_0.4_]O_2_ as both negative and positive electrodes. The greatest advantage of using the same materials as both negative and positive electrodes is to significantly decrease the material's processing cost. Preliminary results show that the full cell delivers an average operating voltage plateau at ∼2.53 V and extraordinary rate (Fig. 4a) and superior cycling performance (Fig. 4b and Supplementary Fig. 7). Even at a very high rate of 12C, the capacity retention is 75% of that at 1C rate. The energy densities of this system are calculated to be 82 and 94 Wh kg^−1^ at current rates of 1C and C/5 based on the mass of positive and negative electrodes. This battery system shows the best performance among symmetric rechargeable batteries reported so far. Although the energy density of this system is not high compared with other reported systems, we can expect that the cost should be much lower than other systems because the abundant elements are used and the same material is used as both positive and negative electrodes. This system may have promising applications in the fields where power capability, cycle life and cost are more critical than energy density, such as large-scale electrical energy storage.

## Discussion

**Figure 5:**
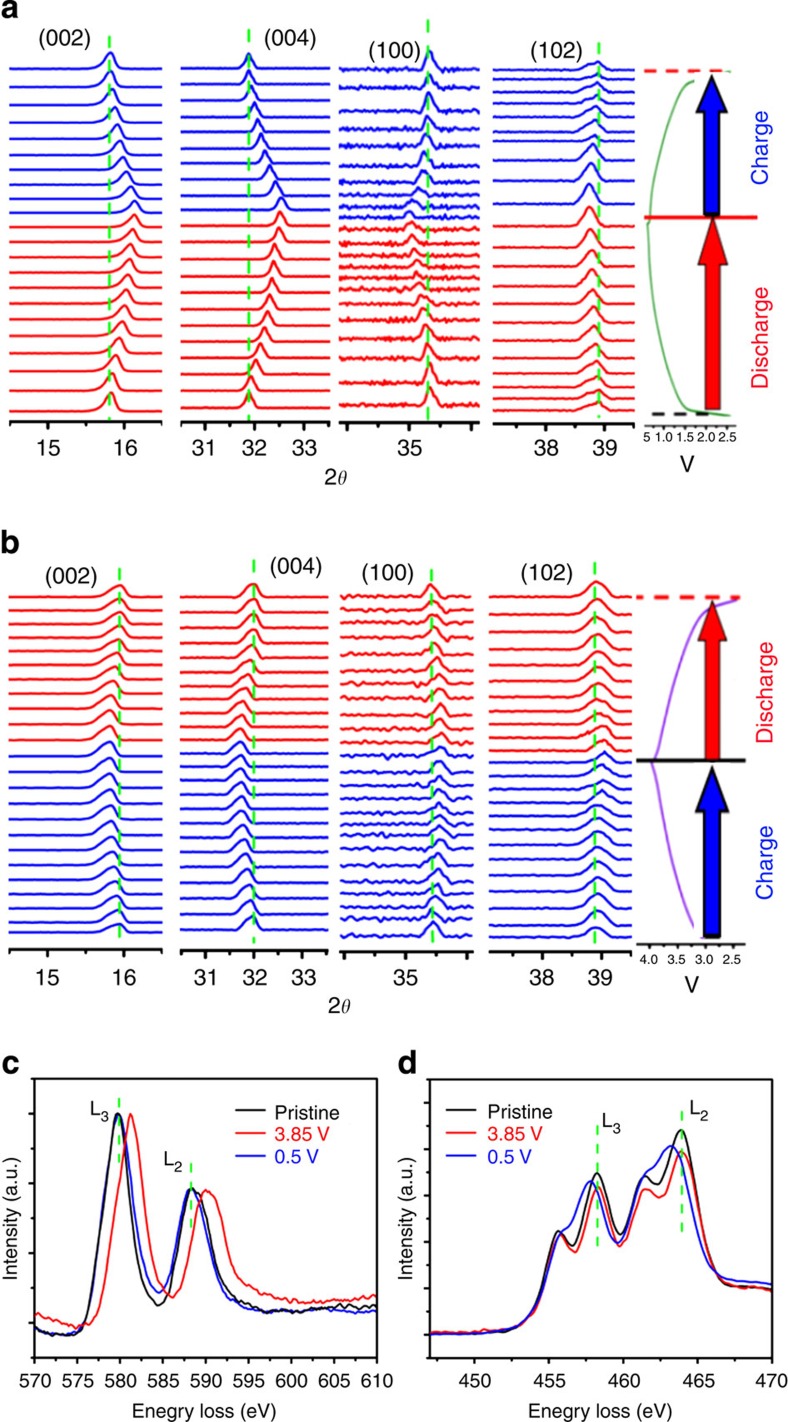
Crystal and electronic structure evolution during electrochemical desodiation and sodiation. *In situ* XRD patterns collected during the first charge/discharge of the Na/Na_0.6_[Cr_0.6_Ti_0.4_]O_2_ cells under a current rate of C/5 at voltage ranges of 0.5–2.5 V (**a**) and 2.5–3.85 V (**b**). EELS for Cr-L_2,3_ (**c**) and Ti-L_2,3_ (**d**) edges for the pristine Na_0.6_[Cr_0.6_Ti_0.4_]O_2_ sample and the Na_0.6_[Cr_0.6_Ti_0.4_]O_2_ electrodes charged to 3.85 V and discharged to 0.5 V, respectively.

To elucidate the structure evolution during sodium deintercalation and intercalation, we carried out the electrochemical *in situ* XRD experiment and the results are displayed in Fig. 5. In Fig. 5a, on Na deintercalation, the (002) and (004) peaks continuously shift to lower two theta angles; while (100) and (102) peaks slightly shift to higher angles, no new peaks beyond P2 structure are observed, indicating a single phase reaction in agreement with the absence of voltage plateaus discussed above. The peak shift indicates that the *ab* plane contracts and the *c* axis expands as expected because of smaller ionic radius of Cr^4+^ and decreasing amount of Na^+^ ions holding transition metal layers together, respectively. The same behaviour is well known for all other materials with brucite-type layers of P2, O3 and P3 structures. In addition, these peak shifts are also highly reversible when Na is inserted back to the structure. A very similar behaviour but with the opposite trend is observed when the Na_0.6_[Cr_0.6_Ti_0.4_]O_2_ electrode was first discharged and then charged as shown in Fig. 5b. For both positive and negative electrodes, the Na storage mechanism is through a single phase reaction, that is, a wide solid-solution exists in Na_*x*_[Cr_0.6_Ti_0.4_]O_2_ in the studied range 0.33≤*x*≤1. In order to investigate the charge compensation mechanism, electron energy-loss spectrum (EELS) and X-ray absorption spectroscopy (XAS) were performed. Figure 5c,d displays the corresponding EELS profiles of Cr-L_2,3_ and Ti-L_2,3_ edges for the pristine Na_0.6_[Cr_0.6_Ti_0.4_]O_2_ sample and the Na_0.6_[Cr_0.6_Ti_0.4_]O_2_ electrodes charged to 3.85 V and discharged to 0.5 V, respectively. In the case of positive electrode, the Cr-L_2,3_ edge shifts to a higher energy while Ti-L_2,3_ edge keeps unchanged on charged to 3.85 V. The shift of Cr-L_2,3_ could be assigned to the formation of Cr^4+^, which is further supported by the XAS results as shown in Supplementary Fig. 8 (refs 44, 45). For the negative electrode, only the Ti-L_2,3_ edge changes significantly, corresponding to the Ti^4+^/Ti^3+^ reduction during Na insertion^46^. These results indicate that Cr^3+^/Cr^4+^ is responsible for the charge compensation on Na extraction, whereas Ti^4+^/Ti^3+^ is involved in the charge compensation during Na insertion, which is in excellent agreement with our initial design.

To confirm that the disordered Cr/Ti distribution in the transition metal layer prevents Na^+^ ordering at any temperature and sodium content such as known to occur in Na_*x*_CoO_2_ (refs 9, 11), we used chemical method to prepare three different compositions, Na_0.33_[Cr_0.6_Ti_0.4_]O_2_, Na_0.5_[Cr_0.6_Ti_0.4_]O_2_ and Na_1_[Cr_0.6_Ti_0.4_]O_2_, and collected high-quality NPD data for those samples at room temperature (Supplementary Fig. 9) and the Na_0.6_[Cr_0.6_Ti_0.4_]O_2_ sample in the wide range of temperature from 3 to 1,073 K. Since, as discussed above, in Na_0.6_[Cr_0.6_Ti_0.4_]O_2_ sodium positions were found to be displaced at room temperature from the centres of trigonal prisms and were expected to be even more disordered at lower Na content and high temperature, the further analysis of the NPD data was performed using not only the Rietveld method but also the Maximum Entropy Method.

**Figure 6:**
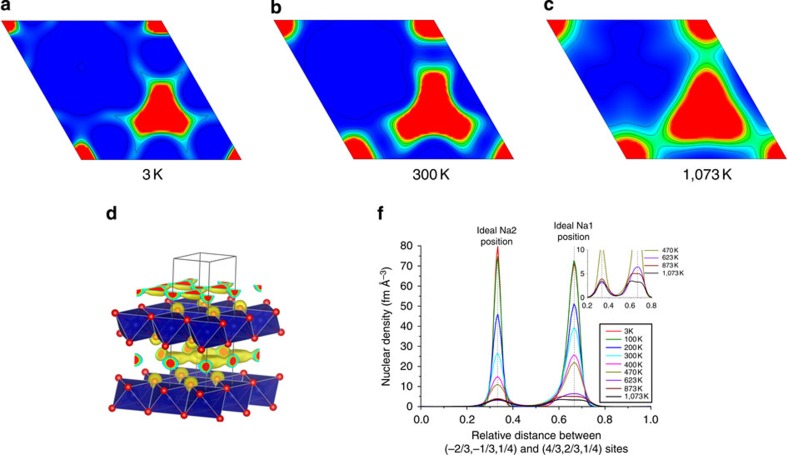
Nuclear density distribution of sodium calculated by the MEM using neutron powder diffraction data. (Top) Section of nuclear density at *z*=0.25 for Na_0.6_[Cr_0.6_Ti_0.4_]O_2_ at 3 K (**a**), 300 K (**b**) and 1,073 K (**c**); (bottom, **d**) general view of the crystal structure with nuclear density at 1,073 K; (bottom, **f**) nuclear density distribution along the line connecting ideal Na1 and Na2 sites as a function of temperature. Inset shows the data at high temperature in greater detail.

The analysis of the NPD data collected for Na_0.6_[Cr_0.6_Ti_0.4_]O_2_ on cooling down to 3 K showed no evidence of Cr/Ti and Na^+^/vacancy ordering such as that found in Na_0.7_CoO_2_ (ref. 11). Instead, the sodium nuclear density was found to gradually delocalize with the increase in temperature (Fig. 6). At high temperature, sodium is disordered into an almost liquid-like state (Fig. 6c) as expected for a material with high ionic conductivity (∼0.6 S cm^−1^ at 1,073 K)^32^. At low temperature the sodium nuclear density becomes more localized, but even at 3 K sodium atoms are still randomly distributed over the Na1 and Na2 sites, and centroids of nuclear density distribution are not in the prism centres (Fig. 6f, inset). On heating from 3 to 1,073 K Na_0.6_[Cr_0.6_Ti_0.4_]O_2_ demonstrates normal Debye-type thermal expansion without anomalies (Supplementary Fig. 10). As expected, the *c* axis expands significantly faster than the *ab* plane as illustrated by linear thermal expansion coefficients estimated in the high temperature range, 22.5 × 10^−6^ and 11.5 × 10^−6^ K^−1^, respectively. Also as expected, the *c* axis expands mostly because of the expansion of sodium-containing interlayer space rather than that of transition metal layers (Supplementary Fig. 10, inset), that in turn further promotes Na^+^ ion mobility.

**Figure 7:**
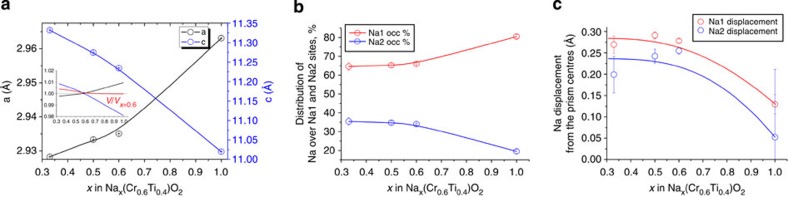
Structure parameters of Na_*x*_[Cr_0.6_Ti_0.4_]O_2_. (**a**) Cell parameters of Na_*x*_[Cr_0.6_Ti_0.4_]O_2_ as a function of Na content at 300 K; inset shows relative change of cell parameters and cell volume with respect to those for the parent Na_0.6_[Cr_0.6_Ti_0.4_]O_2_ material; (**b**) Na^+^ ion distribution over Na1 and Na2 sites as a function of *x*; (**c**) Na1 and Na2 displacement from the centres of prisms as a function of *x*.

The NPD data analysis results for Na_*x*_[Cr_0.6_Ti_0.4_]O_2_ as a function of *x* were found to be consistent with the *in situ* XRD data. The lower the content of sodium is, the shorter *a,b* axes and longer *c* axis are, because of reduction in the average octahedral cation radius and decreasing number of sodium cations holding octahedral layers together, respectively (Fig. 7a). An important consequence of such a combination of the unit cell contraction and expansion is that the unit cell volume changes with *x* very slightly, within 0.5% (Fig. 7a, inset). Furthermore, compared with the parent material Na_0.6_[Cr_0.6_Ti_0.4_]O_2,_ sodium-rich and sodium-poor compositions have slightly smaller and larger cell volume, respectively. This means that changes in the total volume of a symmetric battery using Na_0.6_[Cr_0.6_Ti_0.4_]O_2_ as both negative electrode and positive electrode will be negligible. This is very different from the commercialized LiCoO_2_/graphite battery system where both electrodes expand on charging.

A close of examination of sodium distribution over the Na1 and Na2 sites revealed expected behaviour. When sodium content decreases the growing interlayer distance and number of sodium vacancies further increase Na^+^ ion mobility and overall Na^+^ disorder, which results in the tendency of Na1 and Na2 sites to both equalize occupancies (Fig. 7b) and further delocalize sodium nuclear density (Fig. 7c). This is consistent with the rate performance. Therefore, lowering of *x* in Na_*x*_[Cr_0.6_Ti_0.4_]O_2_ has an effect similar to increasing temperature (Fig. 6a–f).

**Figure 8:**
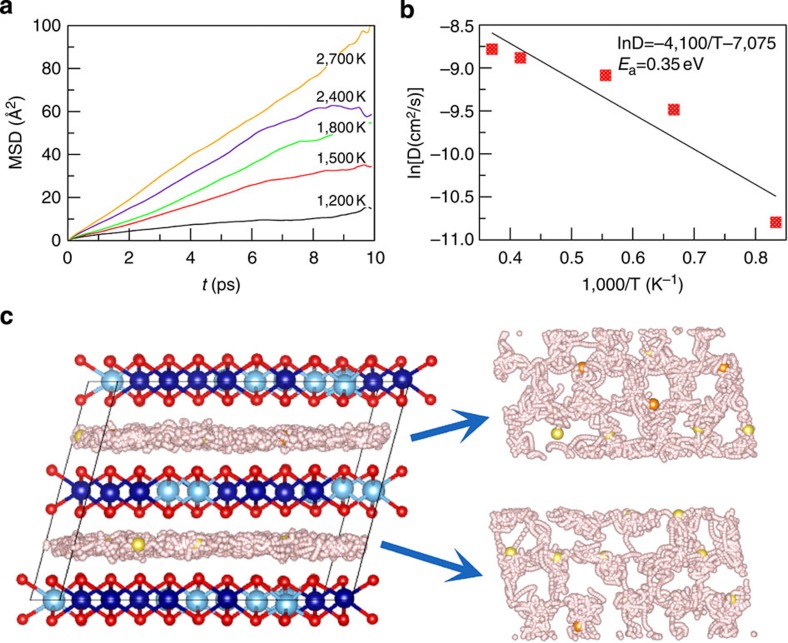
Na^+^ ion transport properties of P2-Na_0.6_[Cr_0.6_Ti_0.4_]O_2_ obtained by FPMD simulations. (**a**) MSD at 1,200, 1,500, 1,800, 2,400 and 2,700 K. (**b**) Arrhenius plot of diffusion coefficients, from which the Na^+^ ion migration energy barrier of 0.35 eV is obtained. (**c**) Trajectories (small grey bullets) of Na^+^ ion in FPMD simulations at 1,800 K from 5 to 8 ps in P2-Na_0.6_[Cr_0.6_Ti_0.4_]O_2_, the top view of each Na^+^ layer is given in the right panel. The initial positions of Na1 (yellow), Na2 (orange), Cr (blue), Ti (light blue) and O (red) are shown for clarity.

To obtain a microscopic picture of the Na^+^ ion transport we also performed first-principles molecular dynamics (FPMD) simulations as implemented in the VASP (Viena *ab initio* simulation package) code^47^. A special quasi-random structure (SQS) was constructed to simulate the disordered configuration of Na^+^/vacancy and Cr/Ti using a 108-atom supercell through the ATAT code^48^. This structure is built from the hexagonal cell with the transformation matrix [−3,−2,0;1,−1,1;0,5,0]. Figure 8 shows the results of FPMD simulations for the P2-Na_0.6_[Cr_0.6_Ti_0.4_]O_2_ system. The mean square displacements (MSDs) of Na^+^ ion for P2-Na_0.6_[Cr_0.6_Ti_0.4_]O_2_ at various temperatures are displayed in Fig. 8a. The obvious Na^+^ ion migration can be observed for all these temperatures. As the temperature increases, the Na^+^ ions diffuse more and more easily. To obtain the diffusion coefficient (*D*), the slope of each curve is fitted in the range from 2 to 8 ps. Figure 8b shows the Arrhenius plot for this material. The diffusion energy barrier (*E*_a_) is extracted from the slope of the Arrhenius plot. The calculated activation energy barrier for P2-Na_0.6_[Cr_0.6_Ti_0.4_]O_2_ is (0.35±0.07) eV, which is comparable to the experimental measurements, 0.24 eV, indicating the effectiveness of our simulations. The detailed diffusion process of Na^+^ ions is shown in Fig. 8c, where the trajectories of the Na^+^ ions are given to illustrate the migration pathways. The positions of Cr, Ti and O atoms are fixed at their initial ones for clarity, since no breaking of Cr–O and Ti–O bonds is found in all the simulations. It is clear to see that the Na^+^ ions migrate within two-dimensional channels, since no diffusion between the Na^+^ layer and the transition metal layer can be found. The top view of trajectories in each Na^+^ layer reveals similar pictures with the section of nuclear density given in Fig. 7. Both of them indicate the pathways along ideal Na1–Na2–Na1 sites.

In summary, through selecting transition metals of Cr^3+^ and Ti^4+^ with very similar ionic radii and a substantial difference in redox potential, we designed a P2-Na_0.6_[Cr_0.6_Ti_0.4_]O_2_-layered oxide simultaneously Cr/Ti and Na^+^/vacancy-disordered in the complete studied wide range of sodium content and temperature. It was demonstrated that this cation-disordered material can function as both positive and negative electrodes with average operation voltages of 3.5 and 0.8 V, corresponding to the redox couples of Cr^3+^/Cr^4+^ and Ti^3+^/Ti^4+^, respectively. A symmetric sodium-ion battery using the same P2-Na_0.6_[Cr_0.6_Ti_0.4_]O_2_ electrode material demonstrates charge/discharge curves free of voltage plateaus, outstanding rate performance and long cycle life. Our study highlights the importance of designing a disordered transition metal/charge arrangement in the layered oxides as a promising strategy to improve their Na-storage performance in terms of rate capability and long cycle stability for the development of high-power and long-life room-temperature sodium-ion batteries.

## Methods

### Synthesis

The resulting material was prepared by a solid-state reaction using precursors of Na_2_CO_3_ (99%), Cr_2_O_3_ (99%) and TiO_2_ (99.5%, anatase form). A phase-pure compound was obtained when an excess of 2 mol% Na_2_CO_3_ was used. The starting materials were ground in an agate mortar and pressed into pellets under pressure of 20 MPa. Then, the pellets were heated at 1,000 °C for 15 h under Ar atmosphere in an alumina crucible.

### Characterizations

The morphologies of the materials were investigated using a scanning electron microscope (Hitachi S-4800). Powder X-ray diffraction was performed using a Bruker D8 Advance diffractometer equipped with a Cu Kα radiation source (*λ*1=1.54060 Å, *λ*2=1.54439 Å) and a LynxEye_XE detector. The XRD pattern was refined using the TOPAS software based on the Rietveld method. In the *in situ* XRD studies, the working electrode was prepared using PVDF as binder on an Al foil. A specially designed Swagelok cell equipped with an X-ray-transparent beryllium window was used for the *in situ* measurements. The *in situ* XRD patterns were collected with an interval of 30 min for each 2*θ* scan from 10° to 80° on charge and discharge at a current rate of C/10, between 0.5∼2.5 and 2.5–3.85 V versus Na^+^/Na. An aluminium foil was placed between the cathode electrode and the beryllium window, to prevent beryllium oxidation at high operating voltages.

The Na_0.6_[Cr_0.6_Ti_0.4_]O_2_ powder was chemically sodiated^49^ by chemical reduction with sodium-biphenyl-1,2-dimethoxyethane (DME) solution as sodiation reagent. In a typical process, 0.0956, g pure sodium was dissolved into 10 ml colourless 1 M biphenyl-DME solution, forming a dark-green organic solution as the sodiating reagent. Then, 1 g Na_0.6_[Cr_0.6_Ti_0.4_]O_2_ powder was immersed into this solution (corresponding to 0.4 mol Na per 1 mol Na_0.6_[Cr_0.6_Ti_0.4_]O_2_). The slurry was stirred until the colour of the solution-fade completely to obtain the sodiated products. The products were washed by DME several times, and dried in the vacuum for overnight. Chemically sodium deintercalation of the sample was accomplished by reaction with a strong oxidant of nitronium tetrafluoroborate (NO_2_BF_4_, Aldrich, 98%)^50^. Solid NO_2_BF_4_ was dissolved in acetonitrile and Na_0.6_[Cr_0.6_Ti_0.4_]O_2_ (molar ratio, NO_2_BF_4_: Na_0.6_[Cr_0.6_Ti_0.4_]O_2_=0.1:1 or 0.27:1) was added to the resulting solution. The slurry was stirred overnight at room temperature, filtered under vacuum and then finally washed several times with acetonitrile.

NPD data were collected using the high-resolution powder diffractometer Echidna at the OPAL research reactor (ANSTO, Lucas Heights) using wavelengths of 1.6215 Å. The NPD data analysis with Rietveld and Maximum Entropy Methods were performed using General structure analysis system^51^ and Dysnomia/RIETAN-FP codes^52^^,^^53^. The crystal structures and nuclear density are visualized with the VESTA 3 program^60^.

EELS spectra were collected with a Gatan GIF spectrometer attached to a JEOL 2010F field emission microscope operating at 200 KeV. The transmission electron microscope was operating at a diffraction mode with a camera length of 8 mm. The spectrometer was set at 0.2 eV per channel with a collection aperture of 1 mm.The EELS spectrum background was subtracted by power law fitting. The electronic states of the Cr during the charge and discharge processes were probed using X-ray absorption near-edge structure spectroscopy techniques at Cr K-edges. The *ex situ* XAS data at the Cr K-edge were recorded at room temperature in the transmission mode at beam line BL14W1 of the Shanghai Synchrotron Radiation Facility (SSRF), China. The station was operated with a Si (111) double crystal monochromator. The photon energy was calibrated with the first inflection point of Cr K-edge in the relevant metal foil. Analysis of the XAS spectra was carried out by using programme code of IFEFFIT^54^.

### Electrochemistry

The working electrode was prepared by spreading the slurry of the active materials (75 wt.%), acetylene black (20 wt.%) and the polyvinylidene fluoride PVDF (5 wt.%) binder on the Al foil. The working electrodes were dried at 100 °C under vacuum for 10 h. The electrolytes are 1 M NaClO_4_ or 0.8 M NaPF_6_ in EC/DEC (4:6 in volume). The coin-type (CR2032) cells were assembled with pure sodium foil as the counter electrode, and a glass fibre as the separator in an argon-filled glove box. The charge and discharge measurements were carried out on a Land BT2000 battery test system (Wuhan, China) in voltage ranges of 0.5–2.5 and 2.5–3.85 V under room temperature. A sodium-ion full cell was constructed using P2- Na_0.6_[Cr_0.6_Ti_0.4_]O_2_ as both the negative and positive electrodes in a 2032 coin-type cell. The weight ratio of the two electrodes (negative/positive) was 1:1.3–1.4 (ref. 55). The full cells were charged and discharged between the voltage range of 1.5–3.0 V at various C-rates (C/10 current rate corresponds to 10.6 mA g^−1^).

### Density functional theory calculations

To simulate the disordered configurations of Na^+^/vacancy and Cr/Ti, the SQS is constructed using a 108-atom supercell through the ATAT code^56^. This structure is built from the hexagonal cell with the transformation matrix [–3,–2,0;1,–1,1;0,5,0]. The FPMD method is adopted to investigate the Na^+^ ion transport properties in P2-Na_0.6_[Cr_0.6_Ti_0.4_]O_2_. The simulations are performed using the density functional theory as implemented in the VASP code^47^,^56^. The present data are obtained using the generalized gradient approximation with a parameterized exchange-correlation functional according to Perdew–Burke–Ernzerhof^57^. The Na(2*p*,3*s*), Cr(3*p*,3*d*,4*s*), Ti(3*p*,3*d*,4*s*) and O(2*s*,2*p*) orbital are treated as valence states. The energy cutoffs for plane wave basis set and augmentation charges are 500 and 756 eV, respectively. The SQS structure is first optimized to obtain the equilibrium lattice parameters and ionic positions on a 3 × 3 × 2 k-mesh. The energy and force convergence criterion for the relaxation are 10^−5^ eV and 0.01 eV Å^−1^, respectively. Then, the FPMD simulations are carried out for 10 ps at each temperature by a Nose thermostat^58^,^59^, and a time step of 1 fs is used to integrate the equation of motion. To keep the computational cost at a reasonable level, only the Γ point is used for the Brillouin zone sampling in FPMD calculations. The MSD can be used to characterize the diffusion behaviour of the system,


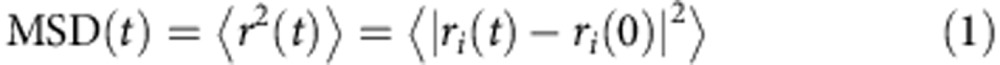


where *r*_i_(*t*) is the position of the *i*-th Na^+^ ion at the time *t*, and the average is over the time steps and all the Na^+^ ions. According to the Einstein equation, the slope of the MSD presents the diffusion coefficient D,


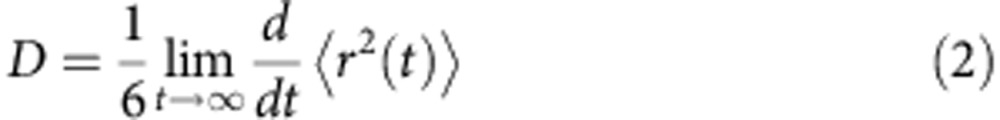


The activation energy barrier for Na^+^-ion diffusion can be extracted from the diffusion coefficients at various temperatures according to Arrhenius equation.

## Author contributions

Y.-S.H. and M.A. designed this work; Y.W. carried out the synthesis and electrochemical experiments; Y.W. performed *in situ* XRD measurements; Y.W. performed *ex situ* XAS measurements, transmission electron microscope and EELS; M.A. collected the NPD data and refined the NPD results; R.X. performed the first principles calculations, Y.-S.H., R.X. and M.A. wrote the paper; all the authors participated in analysis of the experimental data and discussions of the results as well as preparing the paper.

## Additional information

**How to cite this article:** Wang, Y. *et al.* P2-Na_0.6_[Cr_0.6_Ti_0.4_]O_2_ cation-disordered electrode for high-rate symmetric rechargeable sodium-ion batteries. *Nat. Commun.* 6:6954 doi: 10.1038/ncomms7954 (2015).

## Supplementary Material

Supplementary Information Supplementary Figures 1-10 and Supplementary Tables 1-3

## Figures and Tables

**Table 1:** Possible ordered combinations in P2-type layered oxides.

**M1/M2**	**Charge**	**Na^+^/vacancy**	**Example**	**Ratio of M1/M2 ionic radii**
Disordered	Disordered	Disordered	Na_2/3_[Fe_1/2_Mn_1/2_]O_2_ (ref. 31)	1
Ordered	Disordered	Disordered	Na_2_[Ni_2_Te]O_6_ (ref. 20)	1.23
Disordered	Ordered	Disordered	X	
Ordered	Ordered	Disordered	X	
Disordered	Disordered	Ordered	X	
Disordered	Ordered	Ordered	Na_*x*_[Mn_*y*_Co_*1−y*_]O_2_ (refs 18, 21, 34; *y*<0.5)	1.15
Ordered	Disordered	Ordered	X	
Ordered	Ordered	Ordered	Na_2/3_[Ni_1/3_Mn_2/3_]O_2_ (ref. 17)	1.30

More materials are listed in Supplementary Table 1. (Note that ‘X' in Example column refers to ‘no example'.)
