# Supplementary material for: P2-Na0.6[Cr0.6Ti0.4]O2 cation-disordered electrode for high-rate symmetric rechargeable sodium-ion batteries
Source: Nat Commun. 2015 Apr 24;6:6954. doi: 10.1038/ncomms7954 (PMC4421853; doi:10.1038/ncomms7954)
Supplement: Supplementary Information — Supplementary Figures 1-10 and Supplementary Tables 1-3 [file ncomms7954-s1.pdf]

Supplementary Figures

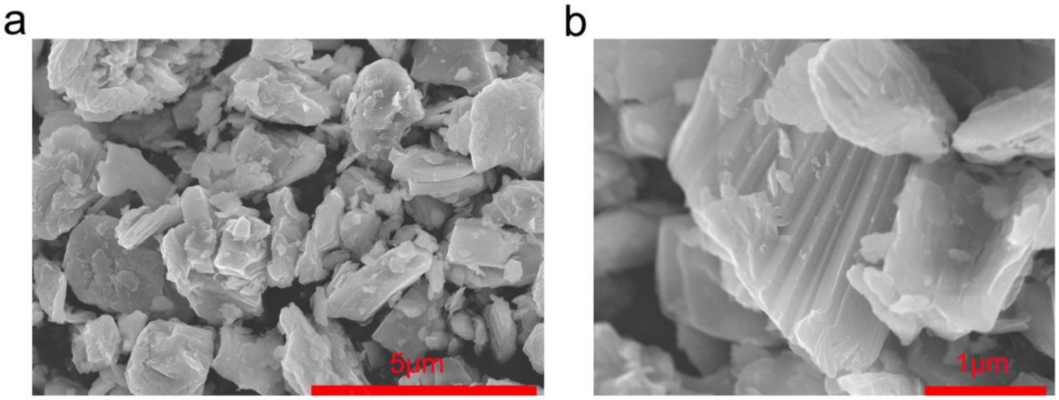

Supplementary Figure 1 | SEM observations. The typical SEM images of the P2-$Na_{0.6}$ $[Cr_{0.6}Ti_{0.4}]O_2$ synthesized by solid-state reaction.

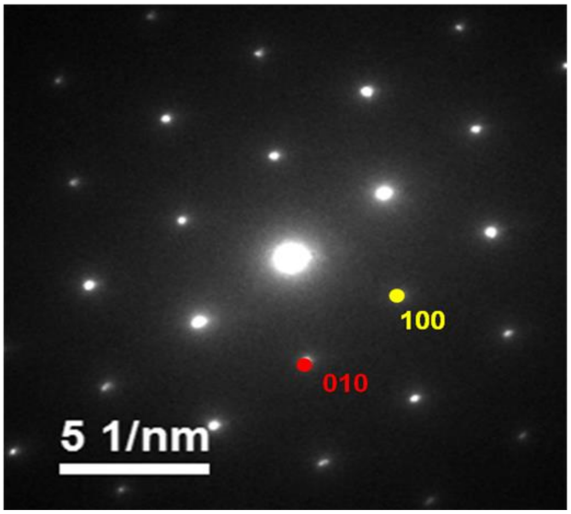

Supplementary Figure 2 | SAED pattern. The typical selected area electron diffraction (SAED) pattern of the P2-$Na_{0.60}[Cr_{0.60}Ti_{0.40}]O_2$ sample.

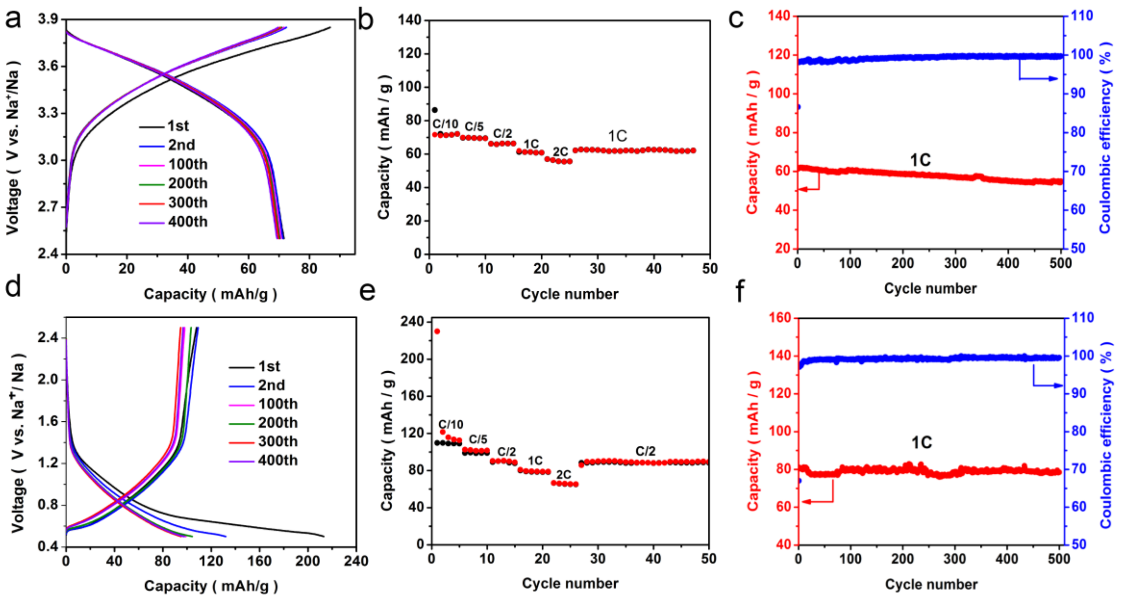

Supplementary Figure 3 | Sodium storage performance of P2-$Na_{0.6}[Cr_{0.6}Ti_{0.4}]O_2$ electrodes cycled in the $NaClO_4$ based electrolyte. (**a**) The 1st, 2nd, 100th, 200th, 300th and 400th charge/discharge profiles at a current rate of C/10 (7.6 mA g^{-1}) in the voltage range of 2.5-3.85 V versus Na^+/Na. (**b**) Rate capability. The capacity versus cycle number at various current rates. (**c**) Long-term cycling performance. The capacity and Coulombic efficiency versus cycle number at a current rate of 1C. (**d**) The 1st, 2nd, 100th, 200th, 300th and 400th charge/discharge profiles at a current rate of C/10 (11.2 mA g^{-1}) in the voltage range of 0.5-2.5 V versus Na^+/Na. (**e**) Rate capability. The capacity versus cycle number at various current rates. (**f**) Long-term cycling performance. The capacity and Coulombic efficiency versus cycle number at a current rate of 1C.

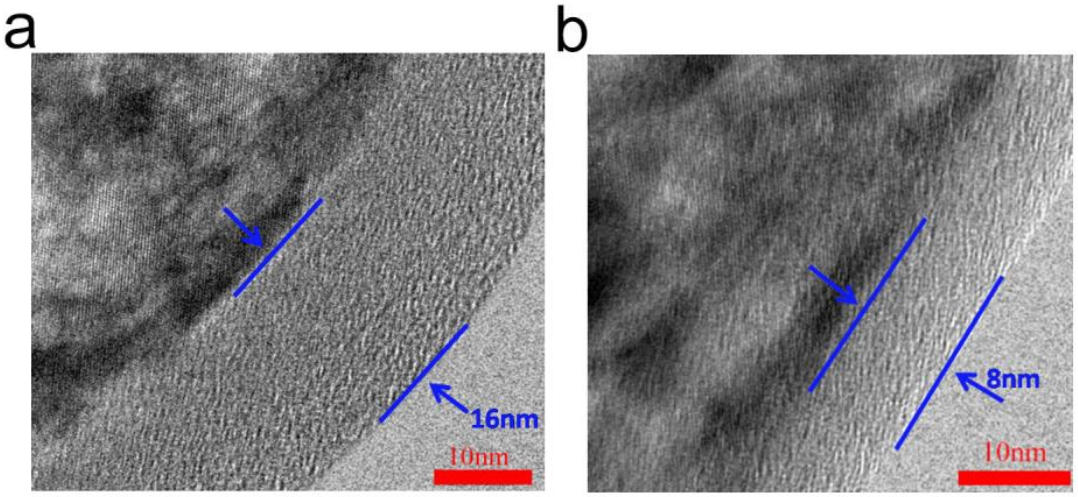

Supplementary Figure 4 | TEM observations. The typical TEM images of the P2-$Na_{0.6}[Cr_{0.6}Ti_{0.4}]O_2$ electrodes discharged to 0.5 V in the $NaClO_4$ based electrolyte (a) and in the $NaPF_6$ based electrolyte (b).

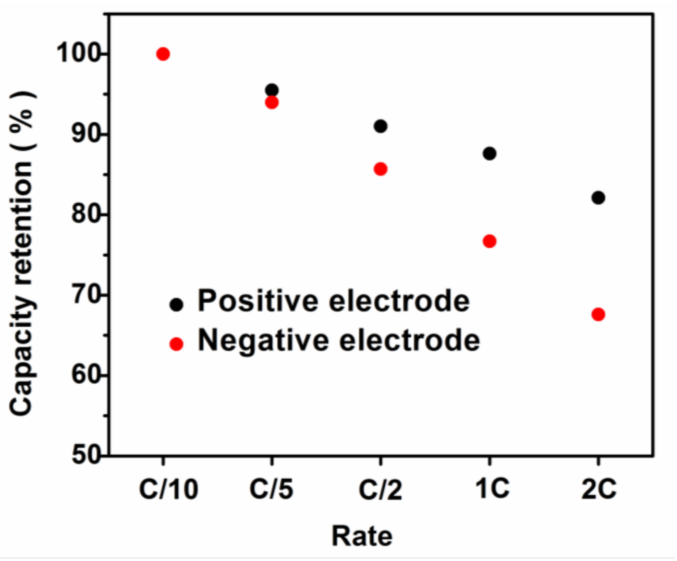

Supplementary Figure 5 | Capacity retention. The capacity retention of P2-$Na_{0.6}$ $[Cr_{0.6}Ti_{0.4}]O_2$ electrodes at current rates of C/10, C/5, C/2, 1C and 2C in the $NaPF_6$ based electrolyte.

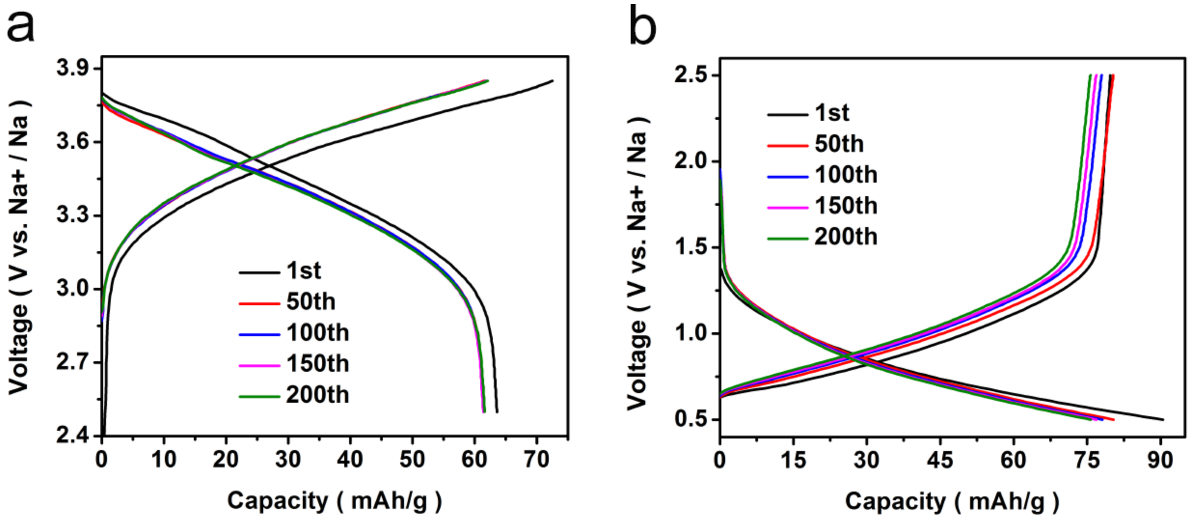

Supplementary Figure 6 | Cycling performance. The discharge/charge curves of P2-$Na_{0.6}[Cr_{0.6}Ti_{0.4}]O_2$ electrodes as positive (a) and negative (b) electrodes cycled at a current rate of 1C in $NaPF_6$ based electrolyte.

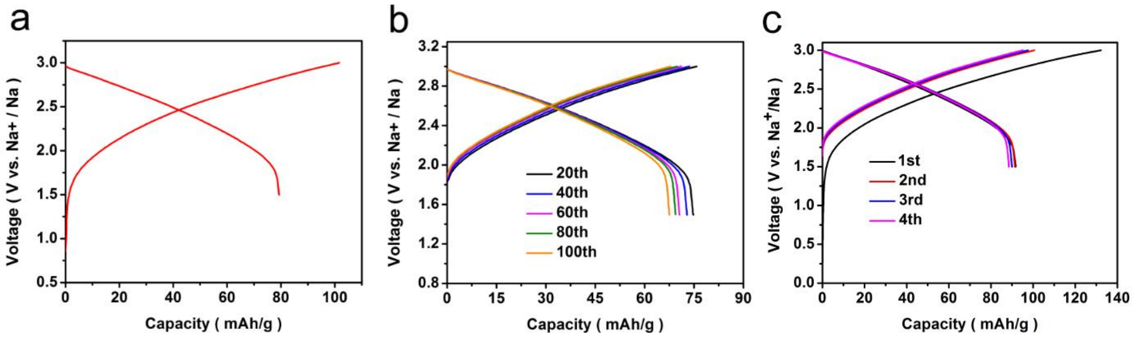

Supplementary Figure 7 | Electrochemical performance of a full cell in the $NaPF_6$ based electrolyte. (**a**) The first charge/discharge profile of $Na_{0.6}[Cr_{0.6}Ti_{0.4}]O_2$//$Na_{0.6}[Cr_{0.6}Ti_{0.4}]O_2$ sodium-ion full cell at a current rate of 1C. (**b**) The typical discharge/charge curves of full cell cycled at a current rate of 1C. (**c**) The typical discharge/charge curves of full cell cycled at a current rate of 0.2C.

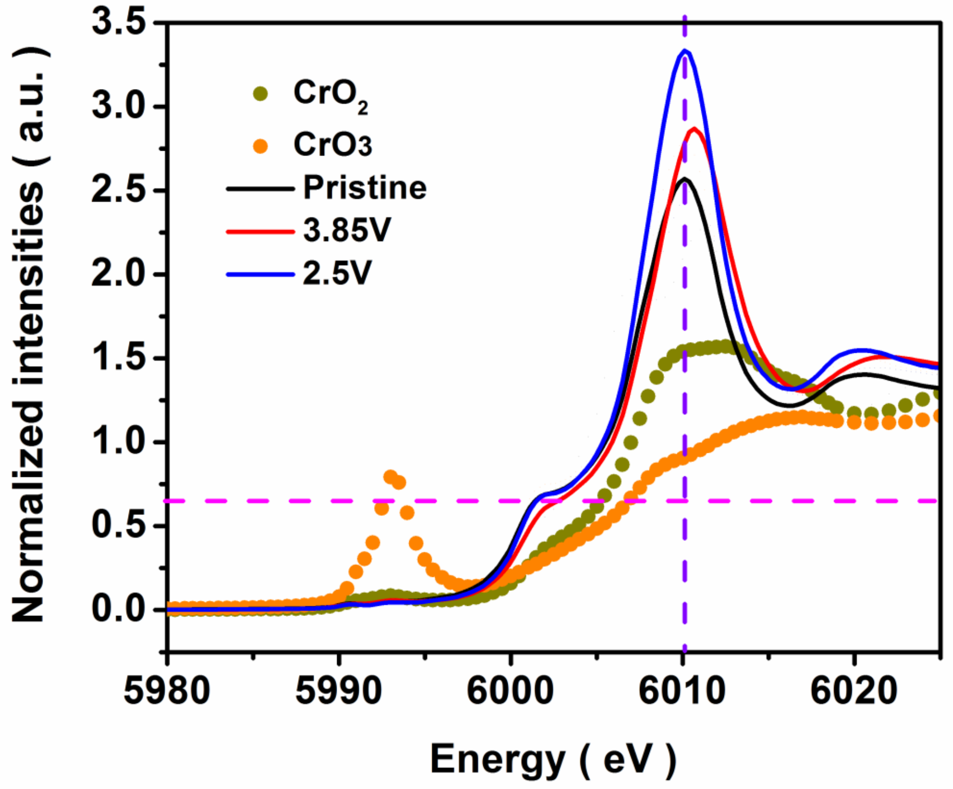

Supplementary Figure 8 | Cr K-edge XANES spectra. The normalized Cr K-edge *ex situ* X-ray absorption near-edge spectroscopy (XANES) spectra of the $Na_{0.6}[Cr_{0.6}Ti_{0.4}]O_2$ electrodes during charge process. The Cr K-edge shifts to a higher energy value upon charged to 3.85 V and shifts back after discharged to 2.5 V. This significant changes indicate that Cr^{3+}/Cr^{4+} is responsible for the charge compensation upon Na extraction. For comparison, the Cr K-edge spectra of CrO_2 and CrO_3 were also shown in the Figure.

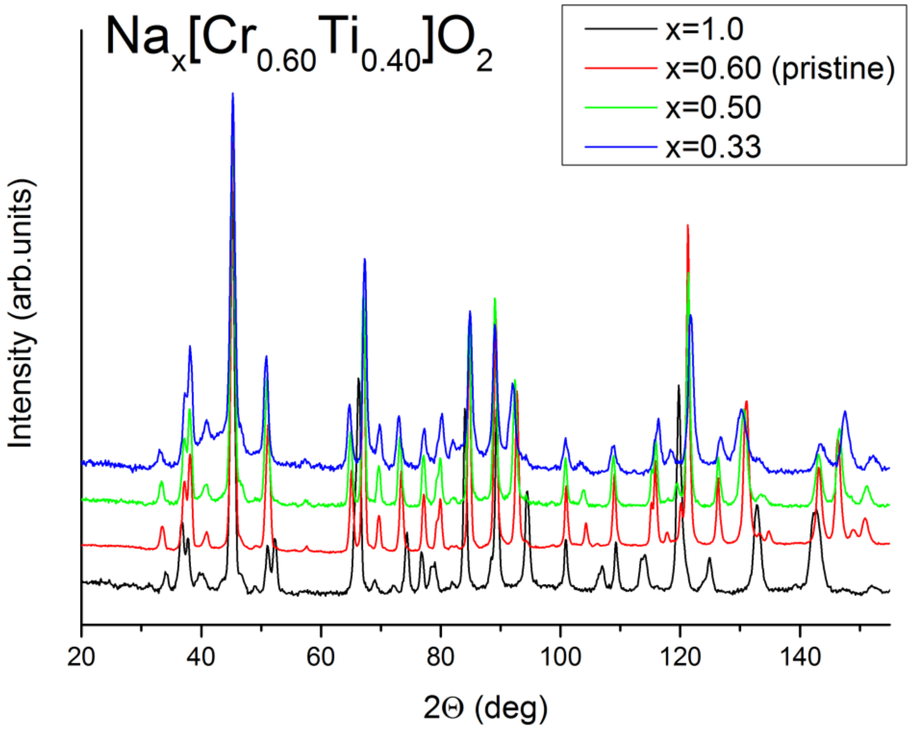

Supplementary Figure 9 | NPD diffraction patterns. Room temperature NPD data for the pristine $Na_{0.60}[Cr_{0.60}Ti_{0.40}]O_2$, chemically desodiated $Na_{0.50}[Cr_{0.60}Ti_{0.40}]O_2$ and $Na_{0.33}[Cr_{0.60}Ti_{0.40}]O_2$ and chemically sodiated $Na_1[Cr_{0.60}Ti_{0.40}]O_2$.

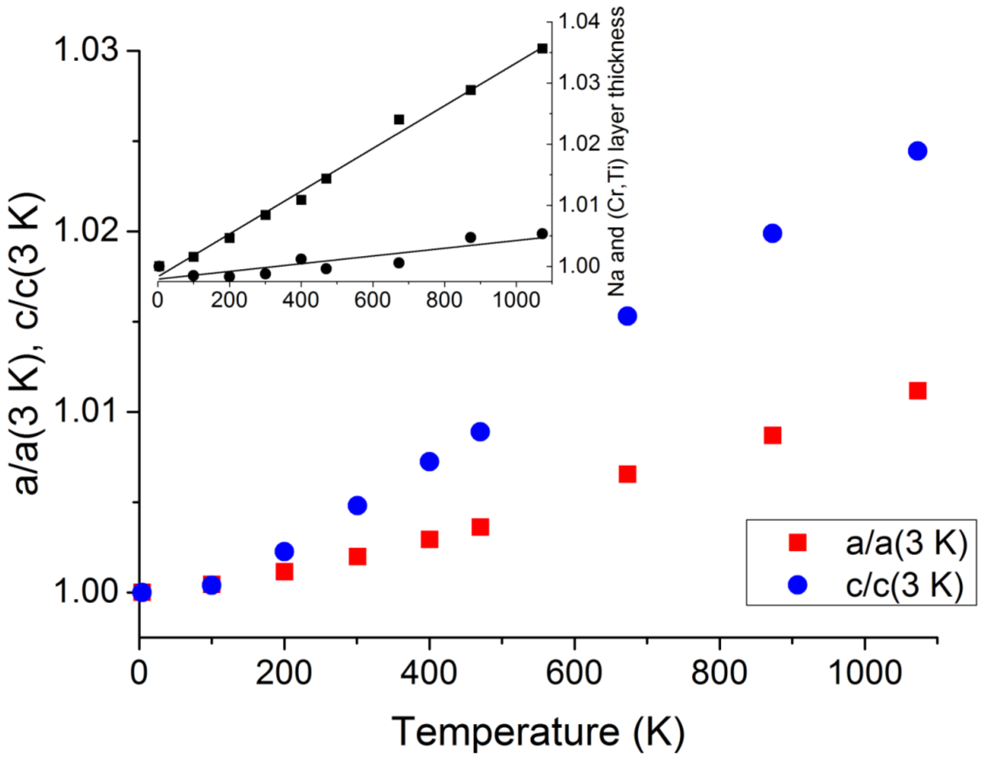

Supplementary Figure 10 | Crystal parameters of different temperatures. Thermal expansion of *a*- and *c*-axes with respect to the cell parameters at 3 K (a=2.93104(5) Å, c=11.1870(4) Å). Inset shows expansion of Na prismatic and (Cr,Ti) octahedral layers with respect to their thickness at 3 K (d(Na) =3.53 Å, d(Cr,Ti) =2.07 Å). Squares and circles in the inset show temperature evolution of the thickness of sodium trigonal prismatic and (Cr,Ti) octahedral layers.

Supplementary Tables

Supplementary Table 1: Disordered/Ordered structure in P2 layered oxides $Na_x[M_1M_2]O_2$

No.	Formula	Ratio of ionic radii	M_1/M_2 ordering	Charge ordering	Na^+/vacancy*	Ref.
1	$Na_{0.74}CoO_2$	1.15		ordered	ordered	9,10,11
2	Na_xVO_2	1.10		ordered	ordered	16,33
3	$Na_{0.6}MnO_2$	1.22		ordered	ordered	13
4	$Na_{5/8}MnO_2$	1.22		ordered	ordered	14
5	$Na_{0.7}MnO_2$	1.22		ordered	ordered	15
6	$Na_{2/3}[Co_{2/3}Mn_{1/3}]O_2$	1.15	disordered	ordered	ordered	18
7	$Na_{0.79}[Co_{0.7}Mn_{0.3}]O_2$	1.15	disordered	ordered	ordered	34
8	$Na_{2/3}[Co_{1/3}Mn_{2/3}]O_2$	1.15	disordered	disordered	disordered	21
9	$Na_{2/3}[Ni_{1/3}Mn_{2/3}]O_2$	1.30	ordered	ordered	ordered	17,35
10	$Na_{2/3}[Ni_{1/3}Mn_{2/3-x}Ti_x]O_2$ ($x<1/6$)#	1.30	ordered	ordered	ordered	25
10	$Na_{2/3}[Ni_{1/3}Mn_{2/3-x}Ti_x]O_2$ ($x\geq1/6$)#	1.14	disordered	disordered	disordered	25
12	$Na_{0.7}[Ni_{0.3}Co_{0.1}Mn_{0.6}]O_2$#	1.30	ordered	ordered	ordered	24
13	$Na_{0.67}[Ni_{0.15}Co_{0.2}Mn_{0.65}]O_2$#	1.13	disordered	disordered	disordered	23
14	$Na_{0.67}[Ni_{0.15}Fe_{0.2}Mn_{0.65}]O_2$#	1.07	disordered	disordered	disordered	32
15	$Na_{2/3}[Ni_{1/3}Ti_{2/3}]O_2$	1.14	disordered	disordered	disordered	28
16	$Na_{2/3}[Fe_{1/3}Mn_{2/3}]O_2$	1	disordered	disordered	disordered	30,31
17	$Na_{2/3}[Fe_{1/2}Mn_{1/2}]O_2$	1	disordered	disordered	disordered	19,31
18	$Na_{2/3}[Co_{1/3}Ti_{2/3}]O_2$	1.07	disordered	disordered	disordered	29
19	$Na_{0.67}[Mg_{0.28}Mn_{0.72}]O_2$	1.12	disordered	disordered	disordered	27
20	$Na_{0.85}[Li_{0.17}Ni_{0.21}Mn_{0.64}]O_2$	1.02	disordered	disordered	disordered	26
21	$Na_{0.66}[Li_{0.22}Ti_{0.78}]O_2$	1.26	disordered	disordered	disordered	22
22	$Na_{0.6}[Cr_{0.6}Ti_{0.4}]O_2$	1.02	disordered	disordered	disordered	
23	$Na_2Ni_2TeO_6$ ($Na_{2/3}[Ni_{2/3}Te_{1/3}]O_2$)	1.23	ordered	disordered	disordered	20

*: In Na^+/vacancy ordering structure, the sodium content is 0.5 in the chemical formula.

#: It can be seen that the content of the third dopant is ≥1/6, then the ordered structure transfers to a disordered structure.

Supplementary Table 2: **Crystallographic data $Na_{0.6}[Cr_{0.6}Ti_{0.4}]O_2$ of with P63/mmc space group**

Chemical formula	$Na_{0.6}[Cr_{0.6}Ti_{0.4}]O_2$
Molecular weight	96.1371
Crystal System, space group	Hexagonal, P63/mmc (No.194)
Temperature	~ 300 K (ambient)
a, b, c (Å)	2.93512(1), 2.93512(1), 11.2342(5)
α, β, γ (º)	90, 90,120
V (Å^3)	83.816(9)
Z	4
Rwp	2.08%
Rp	2.73%

Supplementary Table 3: **Fractional atomic coordinates, occupancies and isotropic displacement parameters**

Site	Wyckoff	x / a	y / b	z / c	occ.	B / Å^2
Na1	6h	0.2823(6)	0.5646(1)	0.25	0.1316	0.0028(1)
Na2	6h	0.0490(1)	0.0980(2)	0.25	0.0684	0.0028(1)
Cr	2a	0.0	0.0	0.0	0.6	-0.0003(5)
Ti	2a	0.0	0.0	0.0	0.4	-0.0003(5)
O	4f	0.6667	0.3333	0.09193(5)	1.0	0.00213
